# Evaluation of large-scale implementation of obstetric point of care ultrasound in eight counties in Kenya using RE-AIM framework

**DOI:** 10.1186/s12913-025-13212-8

**Published:** 2025-08-01

**Authors:** Grace Githemo, Anthony Wanyoro, Jacob Masika, Lister Onsongo, Sarah Bett, Stephen Githuku, Grace Gachuiri, Dilys Walker, Nicole Santos, Rakesh Ghosh, George Otieno

**Affiliations:** 1 School of Health Sciences, Kenyatta University, Nairobi, Kenya; 2 Institute for Global Health Sciences, University of California, San Francisco, USA

**Keywords:** Obstetric, Point of care, Ultrasound, RE-AIM, Implementation science, Kenya

## Abstract

**Background:**

Obstetric Point-of-Care Ultrasound (O-POCUS) holds promise for strengthening maternal health services particularly in low- and middle-income countries (LMICs). However, its widespread use is hindered by limited provider training and resource constraints within health facilities. To address this gap, a large-scale O-POCUS program was implemented across eight counties of Kenya whereby 468 healthcare providers (HCPs) from 224 facilities were trained in five basic O-POCUS parameters. This study evaluated the reach, effectiveness, adoption, implementation, and maintenance of this program using the RE-AIM framework.

**Methods:**

For this cross-sectional evaluation study, trained research staff conducted surveys and in-depth interviews with HCPs, stakeholders, and antenatal and postnatal care clients for one week from a random sample of about half of these facilities (*n* = 114) six months after O-POCUS introduction. A total of 249 HCPs, 2,292 antenatal and 1,704 postnatal clients were surveyed, and 96 HCPs/stakeholders and 114 clients were interviewed. Data were analyzed using descriptive and thematic methods and mapped onto the RE-AIM framework to assess program implementation.

**Results:**

The findings revealed that O-POCUS was implemented across all 114 health facilities and 1937 (49%) of surveyed clients received a scan (reach). Over 80% of trained HCPs reported moderate to high confidence in performing key obstetric assessments, and 72% reported that O-POCUS influenced clinical decision-making including referrals (effectiveness). 41% of HCPs conducted more than 20 scans per month and 89% of the clients reported that they were likely to recommend O-POCUS to others (adoption). Lack of resources such as gel and paper towels were identified as major challenges (implementation), while 60% of HCPs reported the need for further training and mentorship (maintenance).

**Conclusion:**

These findings demonstrate successful large-scale implementation of O-POCUS in Kenya and provide valuable insights for policymakers and healthcare organizations seeking to implement similar O-POCUS programs in resource-limited settings. Continuous strengthening through mentorship, supportive supervision and resource provision is recommended for sustained success of O-POCUS in improving maternal healthcare.

**Supplementary Information:**

The online version contains supplementary material available at 10.1186/s12913-025-13212-8.

## Background

Globally, obstetric ultrasonography is a recommended component of antenatal care (ANC) [1], with the World Health Organization (WHO) recommending that every pregnant woman receive at least one ultrasound before 24 weeks of gestation [2]. Obstetric point-of-care ultrasound (O-POCUS) is a portable technology that can be utilized at the bedside to improve maternal health services including quality and safety of patient care through reducing time to diagnosis, improving diagnostic accuracy, and maximizing procedural safety [3–8]. O-POCUS has also been shown to be beneficial in a resource-limited maternity triage setting in improving midwives’ diagnoses and clinical decision-making at the time of delivery [7, 9–11].

Whereas routine ultrasound screening during pregnancy is a standard practice in most high-income countries, access to this technology for pregnant women in low- and middle-income countries (LMICs) is limited [12]. Poor access to this technology can be attributed to lack of human resources in LMICs with insufficient radiologists or sonographers, thus hampering the provision even in tertiary facilities. Additionally, distance to facilities, cost of ultrasound machines and services, and potential cultural factors further complicate access and uptake [12].

In Kenya, access to O-POCUS services is limited among women seeking ANC especially in rural settings. Only controlled, small-scale studies have shown that O-POCUS provision by trained midwives could improve diagnosis and management of pregnancy [13–15]. Several of these studies have been conducted in urban tertiary hospitals or academic settings with limited numbers of trained providers.

To better understand key facilitators and barriers to scaling O-POCUS including non-tertiary rural settings, Kenyatta University, in collaboration with county leadership teams, the Global Ultrasound Institute (GUSI), and Butterfly Network, implemented a large-scale O-POCUS training and deployment initiative across 224 facilities in Kenya in 2022. This study evaluated the reach, effectiveness, adoption, implementation, and maintenance of this program using the RE-AIM framework. Specifically, we sought to answer the following research questions:

- To what extent did the O-POCUS program reach its intended facilities, providers, and clients?
- How effective was O-POCUS in enhancing clinical decision-making?
- What factors influenced the program’s adoption, implementation fidelity, and long-term sustainability in real-world settings?

Findings from this evaluation is expected to provide insights to guide policy formulation and improve future O-POCUS integration efforts in LMICs.

## Methods

### Study design

A multi-methods approach was employed to evaluate the implementation of O-POCUS at varying levels of the healthcare system and to explore perceptions among pregnant women. The study was guided by the RE-AIM framework which allowed for identification of the barriers and facilitators to successful implementation of the intervention using a structured approached [16].

Data were collected six months post-training of health care providers (HCPs) and after deployment of POCUS devices. As described elsewhere [13], participants underwent a 5-day training focused on five selected maternal and fetal risks – fetal heart rhythm abnormalities, mal-presentation, placenta position, poly- and oligohydramnios, and multiple gestation. Training was facilitated by GUSI while Butterfly Network provided each participant with a Butterfly + probe and an iPad.

### Study setting

Data were collected from predominantly rural health facilities at levels 2, 3 and 4 from eight counties in Kenya: Baringo, Kakamega, Kilifi, Kitui, Nakuru, Samburu, Taita, Taveta and Turkana. These counties were selected because of their high maternal and neonatal mortality and morbidity rates [17]. About 15 counties account for 98.7% of the total maternal deaths in Kenya, attributed to poor access and low uptake of skilled care [18]. Across ten USAID-designated high priority counties, 58% of the mothers deliver at a health facility compared to the national average of 66% [17]. Seven of eight study counties in which O-POCUS was introduced (Baringo, Nakuru, Kilifi, Kakamega, Kitui, Samburu, Turkana) are USAID high priority counties.

### Sampling techniques and sample size

A facility-based sampling method was used to select the study participants for surveys and interviews. We sampled Level 2–4 facilities from each of the eight counties to ensure that county-specific similarities and differences could be elicited. Table 1 lists the number of facilities, providers and clients selected for participation in the study by county. Sample sizes and power calculations were not needed for this evaluation due to its descriptive nature.

**Table 1:** Distribution of study participants by county: health care providers (HCPs), antenatal and postnatal care clients (ANC/PNC)

County	Quantitative surveys				Qualitative interviews			
	Health facilities	HCPs	ANC clients	PNC clients	County stakeholder*	Sub-county stakeholder**	HCPs	ANC clients
Baringo	10	30	228	112	1	1	10	10
Samburu	6	13	113	75	1	1	10	6
Kakamega	25	58	338	318	1	1	10	25
Nakuru	32	48	626	516	1	1	10	32
Kitui	12	37	276	144	1	1	10	12
Taita Taveta	8	15	175	131	1	1	10	8
Kilifi	7	26	318	262	1	1	10	7
Turkana	14	22	218	146	1	1	10	14
**Total**	**114**	**249**	**2292**	**1704**	**8**	**8**	**80**	**114**

*County Reproductive Health coordinator or medical services director; **Facility-in-charge

First, approximately half of all the Level 2–4 health facilities in a county enlisted in the project implementation (114 of 214) were selected using simple random sampling. Subsequently, within each sampled facility all the HCPs who had been trained on POCUS as described above [13] were recruited to participate in surveys; this included midwives, nurses and clinical officers (*n* = 249). All ANC and postnatal care (PNC) clients who received services at the sampled facilities during the five days of data collection were invited to participate in surveys. Utilizing this convenience sampling method, ANC/PNC sample sizes varied depending on facility volume. Ten Level 5 facilities were excluded from this evaluation because they were involved in the initial program implementation.

For qualitative interviews, 10 HCPs were invited from each of the eight Counties (*n* = 80), as well as two key stakeholders per County (*n* = 16). Key informants included facility managers and County management team to understand their perceptions on the introduction of O-POCUS. For client perspectives, one client who had undergone POCUS in each facility was invited to participate in an interview (*n* = 114). These pre-determined sample sizes for the qualitative component were adequate to reach theoretical saturation, while allowing for potential differences between county stakeholders and HCPs to be elicited.

### Data collection

A total of 114 research assistants were recruited for data collection in the eight counties. The research assistants had knowledge of maternal care and were conversant with the culture and local languages for ease of communication and to encourage study participation. They underwent a two-day training to orient them to the data collection tools, ethical procedures around data collection, and standardized data collection guidelines. The training also introduced them to the Open Data Kit (ODK) mobile data collection software. These research assistants conducted all survey data collection, while the project investigators (GG, AW, JM, LO, SB, SG, GG, GO) conducted all in-depth interviews per their assigned county.

Data tools included a facility checklist, HCP questionnaires, client questionnaires, in-depth interview guides for the HCPs and key informants, and an exit interview guide for clients (Appendices 1–7). All study tools were developed by the project investigators based on the components of the RE-AIM framework in order to capture the indicators that were being investigated as defined in Table 2. To ensure the feasibility and reliability of data collection tools, all survey instruments, interview guides, and facility checklists underwent pre-testing and piloting in four facilities in Kiambu County. These pilot sites were not included in the evaluation sample of facilities, but had some HCPs who had been trained on O-POCUS as part of the program. Refinements were made based on pre-testing feedback, improving clarity and usability.

During a 5-day period, research assistants collected data from each facility. All HCPs who consented in the sampled facilities completed the interviewer-administered questionnaires and a subset were invited to participate in the in-depth interviews. All PNC clients attending the postnatal clinic at six weeks post-partum or in the postnatal ward and ANC clients attending the clinic during the week of data collection were invited to participate in the exit surveys. Research assistants ensured clients were appropriately consented and administered the questionnaire only once during the 5-day period. ANC and PNC clients who had undergone O-POCUS were randomly selected to participate in an in-depth interview to help clarify their views, attitude and perception around the use of this new service. Selection of individuals to participate in interviews was based on respondent and research assistant availability and workload.

Lastly, for county-level data (e.g., number of existing facilities) and information related to number of trained individuals and devices deployed across all 224 Level 2–4 facilities included in O-POCUS training, these data were provided by county administrators and GUSI, respectively.

**Table 2:** RE-AIM domains and indicators

Dimension	Definitions	HCP indicators	Client indicators	Data Sources
**REACH**	Extent to which the intervention reaches the target population	● Number of HCPs who were trained and received POCUS ● Proportion of facilities receiving devices per county ● Number of facilities that received devices that currently use them	● Proportion of ANC and PNC clients who received POCUS	● HCP survey ● ANC/PNC client survey ● Facility checklist
**EFFECTIVENESS**	Impact of the program on defined outcomes	● Confidence in ability to identify the five clinical parameters ● Proportion of HCPs reporting O-POCUS impact on referrals ● HCPs perceptions on O-POCUS impact on clinical decision-making	● Proportion of ANC who attended ANC before 24 weeks’ gestation ● Clients perception of O-POCUS impact on clinical decision making	● HCP survey ● ANC/PNC client survey ● HCP interviews ● ANC/PNC client interviews
**ADOPTION**	Extent to which the intervention was accepted by HCPs and clients as part of routine ANC	● Frequency of O-POCUS use by HCPs ● Perceived O-POCUS demand/change in ANC attendance	● Reported satisfaction with O-POCUS scan ● Willingness to recommend O-POCUS services to others	● HCP survey ● HCP interview ● ANC/PNC client survey
**IMPLEMENTATION**	Extent to which the program was implemented as intended	● HCP perceived fidelity in providing O-POCUS ● Payment for O-POCUS services ● Teamwork among HCPs ● Implementation facilitators ● Implementation barriers		● PNC clients surveys ● HCP survey ● HCP interviews ● Facility checklist
**MAINTENANCE**	Mechanisms to ensure sustained use of intervention, including recommendations for use in other settings	● Sustainability of O-POCUS training ● Resource requirements		● HCP interviews ● HCP survey ● Key informants interviews

O-POCUS (obstetric point-of-care ultrasound); ANC (antenatal care); PNC (postnatal care); HCP (health care provider)

### Data management and analysis

Quantitative data were collected using ODK mobile platform. Missing data were addressed through completeness checks during data collection. Data were downloaded into Microsoft Excel, cleaned and exported to SPSS version 26 for analysis. Quantitative data were summarized using descriptive statistics. Missing data were excluded from denominators and analyses when necessary. Qualitative interviews were audiotaped, transcribed and analyzed through thematic analysis. NVivo™ Version 14 software was utilized to manage and analyze qualitative data, with inter-coder agreement checks performed at regular intervals. The thematic analysis involved iterative readings of the quotes for a deeper understanding of the content, labels were assigned to the identified codes and excerpts corresponding to each code were highlighted. Categories were then formulated based on the trends observed from the data. The categories were further scrutinized to merge or eliminate as required and then data was organized into themes and subthemes within the RE-AIM framework [19]. Qualitative data were primarily used to complement or provide additional nuance to quantitative findings, when appropriate.

### Ethics statement

The study was approved by the Kenyatta University Ethical Review Committee (KUERC) number PKU/2563/1689. The study received permission from each of the county research committees. Participants provided informed consent to participate in the study. Confidentiality and privacy were maintained throughout data collection and analysis by ensuring anonymous collection of data and only allowing access to data by the investigating team.

## Results

### Demographic characteristics of the health care providers (HCPs)

A total of 249 HCPs participated in the surveys. The greatest number were from Kakamega County and from level 4 facilities. Most HCPs were aged between 35 and 44 years and were female. The majority were nurse midwives with less than 9 years of experience (Table 3).

**Table 3:** Demographic characteristics of the health care provider (HCP) survey respondents (*n* = 249)

County of employment	*n*	%
Kitui	37	14.9
Taita Taveta	15	6.0
Kilifi	26	10.4
Turkana	22	8.8
Samburu	13	5.2
Kakamega	58	23.3
Nakuru	48	19.3
Baringo	30	12.0
**Health facility level**	**n**	**%**
Level 2	20	8.0
Level 3	100	40.2
Level 4	129	51.8
**Age Group**	**n**	**%**
25–34 years	98	39.3
35–44 years	101	40.6
45–54 years	45	18.1
55 years and above	5	2.0
**Gender**	**n**	**%**
Female	159	63.9
Male	90	36.1
**Cadre**	**n**	**%**
Nurse/Midwife	182	73.1
Clinical officer	40	16.1
Radiographer/Sonographer	13	5.2
Physician	14	5.6
**Years of experience as a HCP**	n	%
1–9	107	42.9
10–19	99	39.8
20 and above	43	17.3
**Years of practice at current facility**	**n**	**%**
1 year and less	52	20.9
2–5 years	125	50.2
6–9 years	35	14.1
Above 9 years	37	14.9

### Demographic characteristics of the antenatal and postnatal clients

A total of 2292 antenatal and 1704 postnatal clients responded to the client survey. Across both ANC and PNC clients the majority attended Level 3 or 4 facilities, were less than 34 years old and were informally employed (Table 4).

**Table 4:** Demographic characteristics of the antenatal care (ANC) and postnatal care (PNC) survey respondents

	Antenatal care clients (*N* = 2292)		Postnatal care clients (*N* = 1704)	
	*N*	%	*N*	%
**Health facility level attended**				
Level 2	262	11.4	256	15.0
Level 3	1069	46.6	733	43.0
Level 4	961	41.9	715	42.0
**Age group**				
15–24 years	1064	46.4	746	43.8
25–34 years	1009	44	760	44.6
35–44 years	217	9.5	193	11.3
45 years and above	2	0.1	5	0.3
**Education level**				
None	243	10.6	145	8.5
Primary	739	32.2	604	35.4
Secondary	885	38.6	684	40.1
Tertiary	425	18.5	271	15.9
**Marital status**				
Single	384	16.8	290	17.0
Married	1884	82.2	1390	81.6
Other*	24	1.0	24	1.4
**Occupation**				
Formal employment	245	10.7	161	9.4
Informal employment	2047	89.3	1543	90.6

*Widowed, did not disclose, separated

The evaluation results are described following the RE-AIM framework and the indicators described in Table 2.

## Reach

Reach represents the extent to which O-POCUS reached facilities, HCPs and clients.

### Device deployment and HCPs trained

For these indicators, we used data available from counties and all implementation facilities (*n* = 224). A total of 468 HCPs were trained and received O-POCUS equipment; ultimately, 467 devices were deployed in 224 facilities across the 8 counties; 214 of these were Level 2–4. These counties have a total of 1622 Level 2–4 facilities, indicating that 13.8% of all eligible facilities received at least one device. Table 5 presents reach indicators for O-POCUS deployment by county. Some facilities had more than one O-POCUS-trained provider and thus received more than one device. Kakamega received the greatest number of devices and Nakuru received the greatest coverage, which ranged from 5.7 to 30.8%. In all counties, coverage was greater in level 3 and 4 facilities than level 2.

**Table 5:** Reach indicators for deployment of O-POCUS by health facility level in each of the 8 counties

County	No. of eligible facilities per county	No. of facilities that received O-POCUS and use them	REACH: % facilities with O-POCUS	# devices deployed per county	# of devices deployed by health level		
					Level 2	Level 3	Level 4&5
Baringo	299	17	5.7	46	0	17	29
Kakamega	281	51	18.1	109	5	47	57
Kilifi	150	14	9.3	40	5	25	10
Kitui	308	23	7.5	63	3	18	42
Nakuru	208	64	30.8	99	12	49	38
Samburu	102	12	11.8	31	0	14	17
Taita Taveta	71	15	21.1	33	1	18	14
Turkana	203	28	13.8	46	8	19	19
**Total**	**1622**	**224**	**13.8%**	**467**	**34**	**207**	**226**

### Clients who received O-POCUS

Among all antenatal and postnatal women surveyed (2292 and 1704, respectively; total 3996), 1937 (48.5%) reported having received an O-POCUS scan. Almost three-quarters of those who received a scan (72.7%, *n* = 1409) attended level 2 and 3 health facilities. Among multigravida PNC clients who had received ultrasound (*n* = 1082), 517 or 47.8% received O-POCUS scan during this pregnancy compared to 24.3% (414) who reported having also received an ultrasound during the previous pregnancy. The majority of scans (74.0%) were performed in ANC clinics, followed by the radiology departments (18%), and then maternity wards (6.0%).

## Effectiveness

Effectiveness describes the impact of the program on decision making and clinical practice indicators. HCPs reported on their level of confidence in identifying the five clinical parameters they were trained on and whether O-POCUS results informed their clinical management or referral decisions.

### Level of confidence in performing O-POCUS

HCPs reported various levels of confidence in identifying the clinical parameters they were trained on (Table 6). Greater than 70% reported feeling extremely confident in assessing fetal position, measuring the fetal heart rate using M mode, measuring fluid to identify polyhydramnios and oligohydramnios. However, HCPs reported feeling less confident in identifying fetal heart rate using audio, multiple gestation, and low lying or placenta. These were also affirmed through the individual in-depth interviews conducted among the HCPs (Table 6, illustrative quotes).

**Table 6:** Level of confidence in performing O-POCUS parameters covered during the initial training (*n* = 249) with illustrative quotes from health care providers

Attributes	Not confident *n* (%)	Somewhat confident *n* (%)	Extremely confident *n* (%)	HCPs perspectives on their confidence in use of O-POCUS
Assessing fetal position (cephalic, breech)	4 (1.6)	36 (14.5)	209 (83.9)	*“I think now*,* I am**** more competent***. *If I can rate myself*,* eight out of ten. I think in terms of competency*,* I think we’ve really learnt a lot and we’ve really improved on our skills.” (HCP 01)* *“As I continue doing it*,* I feel it is becoming* ***automatic and easy***. *So*,* it is like I have advanced.” (HCP 07)* *“As I do the POCUS every day at**** least my skills have improved***. *At least I can be able to identify a multiple pregnancy.” (HCP 20)* *“The**** skills have improved***.*” (HCP 82)*
Measuring fetal heart rate using M mode	5 (2)	49 (19.7)	195 (78.3)	
Measuring fetal heart rate using fetal audio	23 (9.2)	62 (24.9)	164 (65.9)	
Measuring amniotic fluid through single deepest pocket to identify polyhydramnios	10 (4.0)	62 (24.9)	177(71.1)	
Measuring amniotic fluid through single deepest pocket to find oligohydramnios	11(4.4)	55 (22.1)	183 (73.5)	
Assessing for more than 1 fetus (multiple gestation)	13 (5.2)	78 (31.3)	158(63.5)	
Identifying the placenta	15 (6)	63 (25.3)	171 (68.7)	
Identifying the low edge of the placenta to find low-lying placenta or previa	18 (7.2)	86 (34.5)	145 (58.2)	

### Impact of POCUS on clinical management or referral decisions

Among all HCPs surveyed, 197 (79.1%) reported that POCUS findings impacted their decisions to refer a client. When asked on where these clients were referred, over 60% reported referral of the clients to a higher-level facility for continued care or for radiological examination. A majority of HCPs reported that O-POCUS informed their decision to proceed with normal delivery. The use of O-POCUS findings to refer or not to refer was affirmed by the qualitative reports from HCPs and from the ANC and PNC clients (Table 7).

**Table 7:** Provider-reported impact of POCUS on clinical management or referral decisions (*n* = 197) with illustrative qualitative quotes from HCP and clients

Among HCP who used O-POCUS, it informed	More than half of the times and always		Half and less than half of the times		Never	
	*n*	%	*n*	%	*n*	%
Referral to higher level facility for continued care	136	69	54	27.4	7	3.6
Decision to refer for radiological examination	123	62.4	70	35.5	4	2
Decision to proceed with normal delivery*	165	84.2	30	15.3	1	0.5
HCP perception of POCUS findings impact on clinical decision making	*“Several I have sent for**** low-lying placenta**** and they (Sonographer) confirm*,* even for**** multiple gestations**** I’ve sent twice and they’ve confirmed.” (HCP 16)* *“We had a mother who had a**** breech presentation**** at maternity and she had come to deliver. So*,* when we took the scan it was breech and she was primigravida. We referred.” (HCP 47)* *“To do ultrasound and go. But now*,* with POCUS*,* where the client comes here and we use the POCUS*,* we can diagnose and we**** avoid the unnecessary referrals***.*” (HCP 47)*					
Clients’ perception of role of POCUS on HCPs decision to refer	*“They have told me to go**** for a big scan because they think I have twins****)…”(ANC 32*) *“The baby is fine…they told me* ***that the head is okay*** *” (PNC 33)* ***“*** *They told me it was* ***breech presentation and therefore I have to undergo cesarean section*** *” (PNC 34)*					
*one response missing and excluded from denominator (n = 196)						

### Proportion of antenatal clients attending clinic before 24 weeks

Clients were asked to indicate when they attended the first ANC visit which was confirmed from the ANC register. Almost two-thirds (65%, *n* = 1494) started their first ANC visit after 24 weeks of gestation, 28% (*n* = 643) between 13 and 24 weeks, while 6% (*n* = 139) had their first ANC visits before 12 weeks; hence, the majority had O-POCUS after 24 weeks of gestation. Among those who received an O-POCUS scan, (*n* = 287) 22.6% received an US before 24 weeks.

## Adoption

Adoption reflects the proportion of HCPs who integrated POCUS as a routine element of ANC care, as well as their perceptions regarding changes in ANC attendance due to increased POCUS demand. Adoption among clients was reported as satisfaction with POCUS scan services, as well as willingness to recommend it to others.

### HCP-reported frequency of O-POCUS scans performed

HCPs reported on their provision of O-POCUS (Table 8). Approximately 41.4% of HCPs conducted more than 20 scans in the three months prior to data collection, while only 6% of HCPs did not report any use of O-POCUS. Additionally, HCPs felt that ANC attendance increased since the introduction of O-POCUS, citing high demand for this service.

**Table 8:** Provider-reported frequency of O-POCUS scans performed (*n* = 249) with illustrative quotes from health care providers

Number of O-POCUS performed	Frequency *n* (%)	Perceptions about O-POCUS demand increasing ANC attendance
None	16 (6.4)	*“Previously*,* there were few ANC clients but since we started performing ultrasound*,* they come from far where those services are not offered and they come here. So*, ***we see there has been an increase in the ANC services***.*” (HCP 07)* *“The* ***ultrasound services are in high demand*** *in my department. Because we see more mothers when they come to our clinic. You find that you can see around 40 per day].” (HCP 12)* *“In fact*,* in my area it has really increased. Because**** most people are coming in from areas where there’s no scanning***, *to come and do the scanning in my facility.” (HCP 23)*
1–20	130 (52.2)	
21–40	60 (24.1)	
41 and above	43 (17.3)	
Total	249 (100)	

### Client satisfaction with O-POCUS services

Overall, ANC clients who received a scan (*n* = 1269) reported high satisfaction with the care they received. Majority of respondents expressed feelings of happiness upon seeing their baby on the screen. The clients rated O-POCUS services as good, specifically commenting that providers explained to them what they were seeing and answered their questions. Additionally, 64.3% of the clients who received O-POCUS expressed that they were extremely likely to recommend this POCUS service to others (Table 9).

**Table 9:** Client-reported satisfaction with O-POCUS services as reported in antenatal care surveys (*n* = 1269) with illustrative quotes from ANC respondents

Level of satisfaction	Frequency	%	Client perception of the services
Not satisfied	16	1.3	*“Their* ***service was good.*** *I was pleased… Yes they explained everything to me and I was happy.” (ANC-06)* ***“I was happy because I have never experienced that with my other children***. *I’m just happy I have never seen a baby in my womb; I have never been done scanning before.” (ANC-25)* ***“But here … I was given the opportunity to view and see how the baby was***. *As the doctor was looking at the baby I was also looking at the baby*,* how the baby is kicking.**** The doctor was considering me in everything that he was doing**** and if I had any questions I was asking. The whole process I was involved in.” (ANC-21)*
Neutral	100	7.9	
Satisfied	737	58.1	
Very satisfied	416	32.8	
**Feelings towards the fetus upon seeing it on the screen**			
Happy	1014	79.9	
Unhappy	5	0.4	
Anxious	84	6.6	
Not sure	94	7.4	
Others	72	5.7	
**Ratings of the services by the clients**			
Poor	13	1.0	
Good	493	38.8	
Very good	434	34.2	
Excellent	329	25.9	
**Likelihood of recommending POCUS**			
Extremely unlikely	23	1.8	
Somewhat unlikely	4	0.3	
Neutral	97	7.6	
Somewhat likely	329	25.9	
Extremely likely	816	64.3	

## Implementation

Implementation describes the extent to which the program was implemented as intended. Provider fidelity in providing O-POCUS, implementation facilitators and barriers for the POCUS services were evaluated from HCP perspectives. Clients were also asked whether they paid for the services which was meant to be free.

### Provider perceived fidelity in providing O-POCUS

HCPs reported ability to use the new technology 6 months following the initial training (Table 10). Almost all HCPs reported ability to turn the machine on and off and adjust the gain and depth. However, they reported a moderate level of confidence in uploading images and using the modules in the Butterfly application for review. The frequency of accessing the Butterfly training modules varied: 75% reported accessing the learning modules (12% reported never using the modules; 12% daily; 39% weekly and 30%, monthly while 6% accessed occasionally or once in a while.)

**Table 10:** Provider-reported fidelity in O-POCUS functions (*n* = 249)

Provider able to	Frequency	%
Turn the machine on and off	246	99
Adjust gain and depth	244	98
Use learning modules in the Butterfly app	187	75
Send/upload images to other providers	154	62

### Payment for O-POCUS services

POCUS services were meant to be implemented free of charge. ANC clients were asked to indicate whether they paid for POCUS services; 167 (13.2%) paid for the services while most (86.8%) did not, as intended. Among those who paid, 71.2% (119) paid between Ksh 1-1000 (>$8) while 24% (40) paid between KSh1001-3000 ($8-$23).

### Facilitators to POCUS implementation

**Fig. 1:**
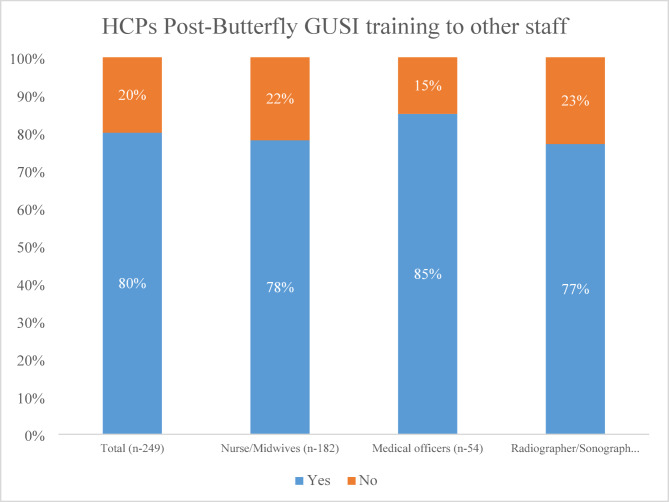
Proportion of health care providers (*n* = 249) who informally trained others on the job (OJT)

HCPs described various ways to expand POCUS implementation. First, each of the HCP cadres trained reported informally training others to use POCUS. Overall, 198 (80%) reported to have trained others through on the job training (OJT) activities (Fig. 1).

HCPs were also asked to indicate whether they consulted anyone in case they were unsure of the O-POCUS findings. Most 93.6% (233) reported to have sought consultation. Most of those who consulted, sought the advice of either midwives or a sonographer.

### Barriers to O-POCUS implementation

Participants were asked to indicate some of the challenges they had experienced in the past month as they implemented O-POCUS. Almost three-quarters of the respondents reported lack of supplies of paper towel, while 60% experienced a lack of gel. Additionally, almost half of respondents mentioned a lack of time to perform ultrasound or faced challenges due to insufficient staffing. These findings were corroborated by the qualitative findings (Table 11).

**Table 11:** Barriers to O-POCUS implementation as reported by health care providers (*n* = 249) with illustrative quotes

Challenge	Frequency	%	HCP reports on the challenges to implementation
Lack of paper towels	176	71	*“Sometimes we use the KY [gel] and we are told that it’s not that good for*,* it’s not friendly with the probe.**** So***,*** we have been raising that issue of gel.**** If you can supply us with the quality gel.” (HCP 68)* *“You might see like 20 and also*, ***there is shortage of staff. Sometimes you are alone and you are running different departments*** *- CWC*,* ANC*,* & FP. So*,* you will**** not be able to scan all the mothers**** who are coming. So*,* you just pick those who are indicated*,* you see if they have a problem.” (HCP 06)* *“Maybe now we want to like sort our images*,* how to upload images; I still have a**** challenge in uploading the images***.*” (HCP 66- clinical officer)*
Lack of gel	150	60	
Lack of time	120	48	
Insufficient staffing	116	47	
Lack of network services	107	43	

## Maintenance

The maintenance dimension of the RE-AIM framework refers to mechanisms to ensure sustained use of POCUS, as well as recommendations for its use in other settings.

### Sustaining O-POCUS training

For the sustainability of O-POCUS services, HCPs were asked the areas they felt they needed further strengthening (Table 12). More than half (60%) of the HCPs reported the need for further support to strengthen their skills related to the five assessments, especially placenta assessment, multiple gestation, and measuring of amniotic fluid. The same was affirmed by the participants during the in-depth interviews.

- *“More training on to identify the gestation*,* and also maybe more practice on locating the placenta.” (HCP 06)*.
- *“Amniotic fluid you know sometimes you get the pouch*,* and you find that it may be a vessel or fetal part there and you cannot measure the pouch. So I am not able to say this is the deepest of the pouches.” (HCP 44)*.
- *“Things to do with placenta*,* presentation. The one that we need more time is the fetal heart*,* amniotic fluid are the sensitive area. But locating multiple pregnancies we are okay.” (HCP 46)*.

Additionally, health facility managers expressed the need to train more staff and provide more training and mentorship to ensure maintenance of the POCUS services.

- *“We need to train as I said everybody that interacts with patients*,* I should be able to walk in into that labor ward and do a POCUS.” (KII-05-Medical superintendent)*.
- *“Yes*,* I have one is capacity building*,* another one is that our rural facilities also need training*,* because most of our referral comes from the rural. We need them to be trained and given POCUS so that they can diagnose early*,* because they refer mother at a late stage when things are not manageable.” (KII-02-Nurse manger)*.
- *“.So*,* if we keep on mentoring people and they take this message positively*,* trust me we will have a positive impact in terms of the utilization of POCUS.”(KII-06 Nurse manager)*.

**Table 12:** O-POCUS parameters that require further training as reported by health care providers (*n* = 249)

Areas requiring further training	Frequency	%
Placenta location	149	60
Multiple gestation	125	50
Amniotic fluid abnormalities	110	44
Fetal presentation	55	22
Fetal heart rate	52	21
None	25	10

### Resource requirements

Both HCPs and managers expressed the need to supply the resources like gel, paper towels and lockable cabinets for the security of the equipment to ensure the sustainability of the services.

- *“What I need for example the provision* ***of ultrasound gel***,*** provision of serviette or tissue paper to cover the mother***,*** and also bundles and all these other things***,*** wipes and the rest. And also***,*** room or coach*** *where I will be performing ultrasound and scanning.” (HCP 86)*.
- *“In the facility*, ***most of our lockers are not lockable***, *they are faulty. And you find the ones*,* which are lockable once they realize there is this machine of 500000 someone will risk and cut that and go with.” (KII-03-KT)*.
- *“Yes*,* it can go on if the county can come in and chip in for the resources that we* ***miss like gel***,*** like the towels***, *like the maintenance of the machine. If the county can come in and incorporate on the use of machine*,* this is something that we can be using in our facility even if the partners can leave.” (HCP 14*).

## Discussion

This study evaluated the large-scale implementation of O-POCUS across eight counties in Kenya using the RE-AIM framework, providing valuable insights into feasibility, scalability, and implementation challenges. Our findings contribute to the growing body of evidence supporting the integration of ultrasound into routine maternal care in LMICs. It highlights the potential benefit from scalability of O-POCUS to enable ANC clients’ access to ultrasound services as per WHO recommendation [2].

Applying an implementation science framework increases our understanding of the challenges and facilitators of ultrasound implementation in real-world settings. Studies have demonstrated that evaluation frameworks, such as RE-AIM, provides a structured approach to evaluating the integration of complex interventions into routine and sustainable use while taking into account contextual factors, existing infrastructure and resources [20, 21]. Indeed, research related to O-POCUS programs in Nepal and Malawi have similarly used the RE-AIM framework to examine barriers to adoption and long-term sustainability in their respective contexts [22, 23]. Like our study, measures were collected through both quantitative and qualitative methods and examined topics such as provider confidence, training and skill maintenance across the five dimensions.

### Reach

We demonstrated that the introduction of O-POCUS increased access to ultrasound services by covering 13.8% of the 1622 eligible Level 2–4 facilities attending to ANC mothers in our selected counties. We showed that the majority of surveyed HCPs, including doctors, nurses and midwives - after a 5-day training - were comfortable using O-POCUS as a component of routine care to screen for five obstetrical parameters. Though not universal, this training was sufficient to achieve a noteworthy proportion of facilities in each county that made O-POCUS available, including lower-level facilities.

While our study demonstrated increased availability of ultrasound services, our findings also suggest that further investment in training, equipment, and policy support is needed to ensure equitable access. With the advancing technology for smaller and mobile ultrasound machines and their lower cost, County governments may eventually be able to procure POCUS equipment as part of their long-term goals for improving prenatal care quality and increase coverage at lower-level facilities. Therefore, more cost-effectiveness studies of ultrasound use in rural settings are needed to justify enhancing O-POCUS reach and availability.

### Effectiveness

Our findings on effectiveness align with previous reports which highlighted the ability to train non-radiology health professionals in LMICS in O-POCUS [11, 13, 22, 24, 25]. Specifically, our findings compare with studies in Uganda, Liberia and Malawi where midwives were trained on ultrasound for a short period of time and were able to diagnose high-risk maternal conditions [22, 26, 27]. Like those studies, most of the trained HCPs in this project were midwives/nurses who are the anchors of the primary health care system in LMICs and the first line of obstetric care.

A systematic review similarly found that short, intensive POCUS training programs are effective in equipping HCPs with essential ultrasound skills, particularly in obstetrics and emergency medicine [28]. In alignment with that review, our study showed that more than three-quarters of the trained HCPs reported high levels of confidence in being able to identify the five high-risk conditions, suggesting that brief but structured training can enhance competency [29]. However, mentorship duration, resource availability, and clinical exposure may play a role in variations in confidence levels, as has been suggested across previous studies in both high and low-income settings [30, 31].

Our effectiveness measures confirm use of O-POCUS impacted HCP decision-making. In addition to providing information to initiate referral, O-POCUS also provided a quick method to exclude findings, which would otherwise lead to unnecessary referral. Ordinarily, women would need to wait for radiologists/sonographers, but using O-POCUS accelerated the process and reduced waiting times. This finding is supported by previous studies where midwives reported that O-POCUS improved their clinical decision-making while providing maternal care [22, 32]. We note, however, that accuracy of antenatal ultrasound for detecting abnormalities shows wide regional variations, with detection rates ranging from 16.7% in Africa to 47.3% in Europe [33]. Our study did not address screening or diagnostic accuracy, emphasizing the need to evaluate training quality and subsequent impact on clinical outcomes.

### Adoption

Although HCPs were largely satisfied with this program, there were notable challenges that impacted adoption. High workload, transfer of trained HCPs and staff shortage were the main health system barriers for the adoption of O-POCUS. When trained providers were absent from work or transferred to another facility, O-POCUS services were interrupted. A study from Rwanda similarly documented the insufficient number of trained HCPs as a barrier to provision of obstetric ultrasound [34]. With few HCPs available at primary care level, adding O-POCUS services on top of routine service provision increased workload to the already overstretched staff. Likewise, studies have shown some resistance to being asked to perform ultrasound examinations without additional re-numeration despite being motivated to [23, 35], a sentiment that was not conveyed in this study. Provision of routine O-POCUS services, therefore, requires training of sufficient number of HCPs on the O-POCUS technology along with assigning additional staff in the ANC clinics and labor wards [36].

Our qualitative findings on adoption are largely in keeping with the published literature, with concerns about inadequate staffing, workload and the migratory workforce [35, 37–39]. Interestingly, to our knowledge, this is the first study to suggest that the introduction of ultrasound may have a specific role in improving team dynamics by improving inter-professional communication and coordination of care. O-POCUS has been shown to be a promising task shifting/sharing approach moving sonographic imaging from radiology departments to frontline healthcare providers [40]. In addition, we report the role of on job training (OJT) in enhancing O-POCUS implementation and sustainability and acceptability by the trained HCPs in passing on knowledge to their untrained colleagues at the workplace. However, further research is required to assess the quality, effectiveness, and sustainability of the OJT.

Despite the modest proportion of women reporting an antenatal ultrasound in our study (48.5%), ANC and PNC clients generally held positive views about O-POCUS services, similar to previous studies on clients’ perspectives and experiences with ultrasound. In a study in Tanzania, no woman declined ultrasound and some reported enjoyment of the ultrasound [43]. A study in a rural Botswana district hospital reported that pregnant women showed signs of trusting the ultrasound results more than their own bodily sensations to confirm a live fetus [42]. Further, most of the interviewed ANC and PNC clients reported that they would recommend O-POCUS services to others, similar to other studies [7, 43]. The dissemination of information about the availability of free O-POCUS services played a great role; clients who had not used ANC services previously were motivated to use the service when they heard about the O-POCUS service availability indicating the need for community awareness and sensitization to increaseuptake.

### Implementation

In general, the intervention was implemented as designed, and there were few challenges reported with POCUS equipment. Supply challenges included shortage of gel, paper towels and a few cases of unstable electricity, as reported elsewhere (37). Unique to this study, plans for remote image review for quality assurance purposes were complicated by HCPs who were unable to update the Butterfly IQ + app for image submission. Additionally, inability to update the app sometimes led to interruption of service provision. Consequently, this caused delays in providing feedback and in some cases made the O-POCUS services unavailable in some facilities. This could partly be mitigated by the use of technology such as WhatsApp which is cheap and readily available and a popular means of communication between HCPs. The use of WhatsApp in healthcare projects is well-established [44] as a way to learn and share ideas; thus, more intentional use of WhatsApp for O-POCUS quality assurance and implementation aligns with the concept of embedding initiatives into pre-existing systems [45] and may warrant further research.

### Maintenance

This study’s findings echo existing evidence for successful O-POCUS machine maintenance beyond use for research purposes in LMICs [46]. To maintain high quality and accurate use of POCUS, the machine must be set with appropriate lighting, temperature, electrical supply and IT requirements [47]. This will require compliance with maintenance protocols [48] and consistent availability of supplies, including gel and supplies for cleaning. Furthermore, easy and robust operability, maintenance service and storage must be thoroughly considered. To ensure local capacity for maintenance, for example, it may also be important to train in-house mechanics to take primary responsibility over minor repairs [48].

Our study results are in agreement with other previous studies which have reported that with adequate training materials, short intensive training courses provide significant acquisition of knowledge and practical skills for all levels of health workers in LMICs (49,50). There is also evidence that follow-up refresher trainings can be effective for retention of knowledge and skills [49, 50] though the sustainability of these courses after donor funding is expended is not adequately documented. However, standardization of training, ongoing education and assessment of practitioners performing POCUS is needed to establish its widespread uptake successfully. Measures such as establishing a national O-POCUS curriculum to carry out crucial short courses for non-radiology/sonographers in collaboration with different specialties of HCPs are imperative. A longitudinal follow up of the performance of the trained HCPs to assess skills retention and routine use of O-POCUS to make clinical decisions as well as evaluate impact of O-POCUS on clinical outcomes and the health care system delivery over time were beyond the scope of this work but highly recommended.

To ensure sustainability, maintenance, quality assurance, and successful implementation of O-POCUS services and programs, focused regulation is needed. The basic ultrasound skills gained by non-radiologist providers are meant to aid them in performing routine clinical duties in the absence of radiologists or sonographers but require regulation to prevent misuse by going beyond the scope of POCUS training. Currently in Kenya, there is no formal regulation or supervision for the O-POCUS non-radiology/sonography HCP trainees outside their formal professional bodies, such as the Nursing Council of Kenya, Kenya Medical Practitioners, Pharmacists and Dentists Council (KMPDU), and Clinical Officers Council of Kenya. These bodies are not mandated to supervise cadres trained in O-POCUS. As O-POCUS continues to be implemented, adequate regulation by professional bodies for non-radiology professionals trained in POCUS is important. For example, issues such as overuse and misuse of O-POCUS, miscommunication between the providers and patients, liability related to incorrect patient diagnosis and care management, and concerns about fetal sex determination remain important areas of investigation and regulation.

### Strengths and limitations

To the best of our knowledge, this is the first study to evaluate large-scale implementation of O-POCUS in in a rural setting with limited health resources in Sub-Saharan Africa. The study included a large sample representing diverse geographic contexts, levels of health facilities and HCP cadres. This study is also among the few that examined the challenges to the implementation of O-POCUS services at primary health facilities in rural Kenya, in contrast to earlier studies that have primarily focused on tertiary settings.

Despite the strengths, the findings of the study should be viewed in light of some limitations. First, the use of purposive sampling for in-depth interviews of HCPs and key informants and convenience sampling for clients limits generalizability. Second, self-reporting of self-confidence and use of O-POCUS may have led to some over reporting, as observed in other LMICs [23, 33]. A more objective assessment, such as independent and concurrent verification of O-POCUS findings, could not be adopted given the core focus was on the implementational aspects rather than on rigorous evaluation of screening accuracy and impact on clinical outcomes. Third, as a cross-sectional study, the findings share the limitations of a descriptive design limiting inferential ability. In addition, the study may or may not represent other clients such as those who sought care at private clinics, or those who received care during the other weeks of the survey month or in other seasons if ANC and PNC trends differ between the week of the survey and other weeks. However, the potential for such differences is small because vast majority of the clientele in rural areas is relatively uniform and consistently utilize public health facilities. Finally, the study’s focus on select counties may also limit generalizability to others because facilities within counties were randomly selected but the counties were not.

## Conclusions

Our study found that large-scale implementation of O-POCUS services to the remote areas of eight counties in Kenya was largely successful and feasible. This model for task sharing among HCPs can be used to increase access to diagnostic imaging in resource-limited settings. However, our findings emphasize the need of refresher trainings and increased mentorship for quality assurance. Shortage of qualified human resources, increased workload, transfers of trained HCPs, lack of POCUS essential supplies must be addressed before transition to scale. Nonetheless, the lessons learnt from this study could provide a policy framework to guide state- and nation-wide implementation of POCUS services, as well as influence similar programs in other LMICs.

## Supplementary Information

Below is the link to the electronic supplementary material.

Supplementary Material 1

Supplementary Material 2

Supplementary Material 3

Supplementary Material 4

Supplementary Material 5

Supplementary Material 6

Supplementary Material 7

## Data Availability

No datasets were generated or analysed during the current study.
