# Supplementary material for: Evaluation of large-scale implementation of obstetric point of care ultrasound in eight counties in Kenya using RE-AIM framework
Source: BMC Health Serv Res. 2025 Aug 1;25:1016. doi: 10.1186/s12913-025-13212-8 (PMC12315356; doi:10.1186/s12913-025-13212-8)
Supplement: Supplementary file 1 — Supplementary Material 1 [file 12913_2025_13212_MOESM1_ESM.pdf]

APPENDIX 1: QUESTIONNAIRE FOR HEALTH CARE PROVIDERS TRAINED ON POCUS

Section 1: Background Information

**Date of visit:**		**Time start**	
ID/Serial # of Ipad/tablet			
Email associated with your probe and tablet			
**County** *(Circle the appropriate response)*	Baringo--------------1<br>Kakamega-----------2<br>Kilifi-----------------3<br>Kitui-----------------4<br>Nakuru---------------5<br>Samburu-------------6<br>Taita Taveta---------7<br>Turkana--------------8	**Sub-County:**	______________________________
**Health Facility Name:**		**Health Facility Level**	
**Facility Type** (*circle the type of facility*)		**Site of Facility** *(Circle the site of facility)*	**1. Urban (define this in the protocol)**<br>**2. Rural**

**Demographic**

		CATEGORY	*CODE*	*SKIP*
Q1	Please select your profession	Physician………………<br>Nurse/Midwife …………<br>Clinical officer …………..<br>Radiographer /Sonographer<br>Others (Specify)	1<br>2<br>3<br>4<br>5	
Q2	What is your age (*in years*)?	________________		
	What is your gender?	Female---------------------<br>Male------------------------	1<br>2<br>3	**Maximum Age is 65**
Q3	How many years have you worked since qualification as a health care worker	_____________________		
Q4	How many years have you worked in the current facility?	-------------Q4<=Q3--		
Q4	a. Which unit/department are you currently stationed?	Antenatal Clinic<br>Family Planning<br>Labor ward<br>Post-natal ward<br>Maternity Ward<br>Other (specify)_have multiple	1<br>2<br>3<br>4<br>5	

	b. How many years have you been posted in the current unit	Q4b<=Q4 or Q3		
	c. Were you stationed in the current unit/department before the butterfly Point of Care US (Butterfly POCUS) training?	Yes<br>NO		If no
		If no, specify…………		
Q6	Have you ever performed a POCUS exam before the butterfly POCUS training?	Yes…………………….<br>No……………………..<br>If no skip to Q*8?…………	1<br>2	----→Q7
	c). Have you ever performed a non-Butterfly probe obstetric ultrasound exam before the POCUS training?	Yes<br>No	If Yes go to Q7<br>If no skip question 7	
Q7	How many obstetric ultrasound exams had you done before POCUS training in your career?	<10(novice)<br>10-50 (learner)<br>>50 (advanced)	1<br>2<br>3	
Q8	Had you used a tablet or smartphone before POCUS?	Yes…………………….<br>No………………	1<br>2	If no go Q10

Q9	How often do you use a tablet or smartphone (touch screen)?	Daily<br>Weekly<br>Monthly<br>Other: (Specify) _____	1<br>2<br>3<br>4	
Q10	Does your facility have capacity to perform caesarian sections operations	If yes go to Q11b………………….<br>No >Q11………………	1<br>2	-----→Q11
Q11	How far are you from a hospital with caesarian section operative capacity (By road in a vehicle?)?	Less than 1 hour -------------<br>1-3 hours ----------------------<br>>3 hours -----------------------	1<br>2<br>3	
11b	Does your facility have Standard Ultra Sound	Yes >Q11c and Q11d<br>No >Q12		
11c	If yes to 11b is it functional	Yes<br>No		
11d	If yes to 11b does the facility have a sonographer/radiologist	Yes<br>No		
	**SECTION 2: GENERAL QUESTIONSCUS** Training and confidence			
Q12	Were you trained on how to perform ultrasound by another organization prior to POCUS (except the one done GUSI- From Sep-Dec 2022?	Yes >Q12a1…………………….<br>No >Q12b	1<br>2	If yes

Q12a1	which organization and when			
Q12a2	when			Not behold 2023
Q12b	Have you been trained on how to perform other ultrasound after Butterfly POCUS training (except the one done GUSI?	Yes…Q12c…………………<br>No >Q13	1<br>2	
Q12c	Which organization trained you?			
Q12d	when?			Not behold 2023
Q13a	After Butterfly GUSI training, have you received any further training on butterfly POCUS?	Yes…Q13b…………………<br>No…Q13d………..	1<br>2	
Q13b	Who gave you further training you?	Have choices for who -----------<br>Radiographer<br>Nurse/ Midwife<br>Doctor<br>Clinical officer<br>Radiologist<br>Person we were trained with<br>Any other.<br><br>For **when** leave it open-------------------		
Q13c	When were youn trained?			
Q13d	Since you were trained to use Butterfly POCUS have you trained any other person on its use	Yes >Q13e<br>No > Q201		

Q13e	How many persons have you trained			
Q13f	What are their cadres.	Persons Cadres<br>Nurse<br>Clinical officers<br>Doctors<br>radiographers		
Q13g	Indicate the department	Medical Department<br>Surgical Department<br>Obstetrics/gyn Department<br>Pediatrics Department<br>Accidents emergency Department		

Q201:How confident do you feel with conducting each of the following? (Ease of use) (Select one box per row) (After POCUS training)

Items	I do not know how	Extremely not confident	Somewhat not confident	Neither confident nor not confident	Somewhat confident	Extremely confident
Q201.1. Turning the machine on and off						
Q201.2. Adjusting gain and depth						
Q201.3. Measuring fetal heart rate using M mode						
Q201.4. Measuring fetal heart rate using Fetal Audio (hearing the heartbeat)						
Q201.5. Assessing fetal position (cephalic, breech)						
Q201.6. Assessing for more than 1 fetus (multiple gestation)						

Q201.7 Sending /uploading images to other providers						
Q201.8.Identifying the placenta						
Q201.9. Identifying the lower edge of the placenta to find low-lying or previa						
Q201.10.Identifyin g amniotic fluid						
Q201.11. Measuring Amniotic fluid to through single deep pocket to find oligohydramnios.						
Q201.12.Measuring fluid to through single deep pocket to find polyhydramnios						
Q201.13.Measuring gestational age using biparietal diameter						

Q201.14. Measuring gestational age using head circumference						
Q201.15. Using the learning modules available in the Butterfly App			.			

Q201a: How confident **are you in conducting other POCUS examination like?**

Items	I do not know how	Extremely not confident	Somewhat not confident	Neither confident nor not confident	Somewhat confident	Extremely confident
**Q201a.1 Gynecologic**						
Q201a.2 Heart ultrasound						
Q201a.3 Musculoskeletal						

Q201a.4 Doppler ultrasound						

Please comment freely on any other factor you consider important and not mentioned in the table

Q202a Is there anything else confusing or frustrating for you when using the ultrasound probe or software?

a) Yes >Q202b
b) No >Q203

. Q202b If yes, please describe what is frustrating or confusing.

Q203 How often do you access the POCUS training modules in the tablet?

- Never (if never skip q203f)
- Daily
- Weekly
- Monthly
- Other, specify

Q203a .Which modules have you accessed?

- Include all modules

- ……..

Q203b Which modules do you think are most valuable?

- Modules…
- ….

Q203c Which modules are missing?

- I don't know
- Modules….

Q203d What ultrasound topic/modules would you like to know more about?

- Modules…
- Others: specify…….

Q203e Which POCUS exams/module do you think will be most useful for you in your future practice?

- Modules….
- Others: specify…..

Q203f Why have you never accessed the POCUS training modules in the tablet?

SECTION 3: UTILIZATION OF POCUS

Q300: Since you were trained to perform POCUS have you ever conducted an US using the equipment

1. Yes > Q301
2. No >Q302

Q301: Is yes, how often (very often, often, rarely, very rarely)
Q302: Reasons why not trained.

Q303: How many POCUS have performed yourself over the **last 3 months?**

◻ 0
(if zero also skip to question Q332),
◻ 1-10, ◻ 11-20, ◻ 20-30, ◻ 31-40 ◻ over 40.
If zero, ask why ?

Q304: Approximately how many POCUS (patients) have you performed yourself over the **past 1 month?**

◻ 0(skip next two questions), ◻ 1-5, ◻ 6-9 ◻10-20 ◻21 -30 ◻ over 30

Q305 If Zero ask Reason Q2b why

Q306 Approximately how many POCUS (patients) have you performed yourself over the **past week?** Now that POCUS is part of your practice.
none (skip next question),
◻ 0(skip next Q309), ◻ 1-5, ◻ 6-9 ◻10-20 ◻21 -30 ◻ over 30

Q307 In the past month, what unit did you perform most of the POCUS scans in?
a) ANC clinic--------
b) Maternity ward-----------
c) Labor ward-----------
d) Others specify--------

Q308Your answer to question 307 above, fill in the number you performed in specific units (Fill where applicable)

e) ANC clinic--------
f) Maternity ward-----------
g) Labor ward-----------
h) Others specify--------

Q309. Have you performed POCUS in any other place over the last 1 month other than in 3 above

1. Yes, >Q310
2. No,> Q311

Q310 If yes, where else have you performed US? ------------------Specify the number per month (if applicable)

Q310a. ANC clinic--------
Q310b. Maternity ward-----------
Q310c. Labor ward-----------
Q310d. Others specify--------

□ 1-5
□ 6-9
□10-20
□21-30
□ Over 30

Q311. How long does it take in minutes to perform an US per patient using the Butterfly POCUS?

- <10
- 10-20,
- 20+
- Depends on the case, specify

Q312 If you have done Ultrasound using Butterfly POCUS, how frequently or infrequently do you think ultrasounds which you performed could help you to decide on a treatment plan for your patient?

- Never (if never skip next question)

- Less than half the time
- About half of the time
- More than half the time
- Always
- Other, specify

Q313 How often have the findings impacted clinical management /influenced your decision making:

- Never
- Less than half the time
- About half of the time
- More than half the time
- Always
- Other, specify

Q314: Have you referred a patient because of a POCUS finding

1. Yes >Q315
2. No>Q319

Q315 How often have the POCUS findings informed decision to refer to higher level facility

Q316 How often have the POCUS findings informed decision to refer for further radiological reviewto higher level facility

Q317 How often have the POCUS findings informed decision to increase frequency of patient follow-up>

Q318 How often have the POCUS findings informed decision to refer proceed with birth?

- Never
- Less than half the time
- About half of the time
- More than half the time
- Always

Q319: If POCUS influenced care modification, indicate the decision that you made

a) Specialist care in your facility
b) Higher level for specialist consultation outside your facility
c) Higher level for CS
d) Radiologist for formal US
e) Monitor for birth
f) Others (Specify)…………………………………

If you have been doing pocus answers the following questions

Q321	Using POCUS have you identified any abnormal cases of fetal heart rate?<br><br>Q322. If yes how many times	Never<br>Yes if yes how many times<br>a. A few times<br>b. Many times	1<br>2	
Q323	Using POCUS ultrasound, have you identified any cases of multiple gestation?	Never<br>A few<br>Many ………………	1<br>2	

Q324	Using POCUS, have you identified any cases of malpresentation (Eg. Breech position of baby)?	Never<br>A few<br>Many ………………	1<br>2	
Q325	Using POCUS, have you identified any other high-risk conditions?	Never<br>A few >Q326<br>Many > Q326	1<br>2	
Q326	What condition(s)?	Multiple gestation<br>Placental abnormalities<br>Amniotic fluid abnormalities<br>Abnormal lie<br>Abnormal foetal heart rate/s<br>Other, specify……..		

Q327 What do you think is your capacity to perform a Butterfly POCUS has on the quality of care (QoC) you offer?

a) Decreased the quality of care,
b) Not changed the quality of care
c) Improved the quality of care
d) Others, specify

Q328 On a scale from 1-5, please rate your level of comfort in acquiring images with the POCUS machine (1= not comfortable at all; 5= very comfortable):

1. Not comfortable at all
2. Not comfortable

3. Neutral
4. Comfortable
5. Very comfortable

Q329. On a scale from 1-5, please rate your level of comfort in **interpreting** each of the following POCUS exams (1= not comfortable at all; 5=very comfortable):

Q329a. Foetal presentation (vertex or breech),	□ 1	□ 2	□ 3	□ 4	□ 5
Q329b. Multiple gestations	□ 1	□ 2	□ 3	□ 4	□ 5
Q329c. Foetal heart rate abnormalities	□ 1	□ 2	□ 3	□ 4	□ 5
Q329d. Placental location (low lying placenta and placenta previa)	□ 1	□ 2	□ 3	□ 4	□ 5
Q329e. Amount of amniotic fluid (polyhydramnios/oligohydramnios)	□ 1	□ 2	□ 3	□ 4	□ 5
Q329f. Gestational Age	□ 1	□ 2	□ 3	□ 4	□ 5

Q330 In your opinion, how have your patients responded to your use of POCUS in ANC?

□ they have responded positively

□ they have been worried/negative about its use

□Neither positively/negatively

□ I am not sure

□Other (specify) ……….

If Maternity unit (labor & delivery) in Q3

Q331. In your opinion, how have your patients responded to your use of POCUS in Labour ward?

□ They have responded positively

□Neither positively/negatively

□ They have been worried/negative about its use

□ I am not sure

□Other (specify) ……….

Q332. Indicate your level of agreement with the following statement where 1 is strongly disagree and 5 is strongly agree- My ability to provide point of care US is useful to my practice

□ Strongly disagree □ Disagree □ Neutral
□ Agree □ Strongly Agree

Q333. Is the POCUS US probe being used for another purpose other than the training you received?
1. Yes >Q333

2. No >Q334

Q333others. if yes specify --------------------------

Q334	Out of the five examinations you were trained. Which do you think was most useful for you in your current and/or future practice? (Tick all that apply)	• Foetal presentation (vertex or breach),<br>• Foetal heart rate,<br>• Placental location, Normal, low-lying placenta and placenta previa<br>• Multiple gestations<br>• Amniotic fluid abnormalities including oligohydramnios.

Q335	What POCUS skills among the ones you were trained on would you like to know more about? (tick all that appy)	• none<br>• Foetal presentation (vertex or breach),<br>• Foetal heart rate,<br>• Placental location, Normal, low-lying placenta and placenta previa<br>• Multiple gestations<br>• Amniotic fluid abnormalities including oligohydramnios.
Q336	Do you consult anyone in case you are not sure of your Butterfly POCUS findings?<br><br>Q336aYES, if yes who do you consult<br><br>Q336b. NO if no what is the reason of not consulting (I don't know whom, I feel discouraged, I ignore)	A midwife<br><br>A radiographer<br><br>A clinical officer<br><br>A radiologist<br><br>An obstetrician<br><br>Other-
Q337	Do you record findings from each scan?<br><br>Yes >Q337a<br><br>No >Q338	
Q337a	Where do you record your finding	Mother booklet<br><br>Mothers file<br><br>A piece of paper

Q337b	How often	All the time<br>sometimes		
Q338	What findings do you record from US findings	Normal……………<br>Abnormal…………..<br>I don’t, there is no place to document.<br>All…..		
Q339a	Since the introduction of POCUS in your facility, has it affected the way you perform clinical routine examination of pregnant mother’s such as palpation of mothers	Yes >Q339b<br>No >Q340a		
Q339b	If it has affected indicated which ones	manual palpation, use of fetoscope, BP monitoring, provision of counseling<br>Others -----		
Q340a	Have you noted improved quality of labour and delivery service since the introduction of POCUS in your facility	Yes >Q340b<br>No > Section 4		
Q340b	If yes specify area have you noted improvement			

Q340c	If yes how has the quality of care been affected	quality of care increased overall,<br>quality of care decreased overall.<br>Others, specify……		

Section 4: Challenges /barriers on utilization on POCUS

Q400. How often in the last one month have you experienced the following problems with your POCUS equipment?

		Never	Rarely	Some of the time	A lot of the time
A	Tablet Malfunction				
B	Probe overheating and stopping				
C	Tablet stolen or lost				
D	Probe stolen or lost				
E	Charger stolen or lost				
F	Lack of electricity				
G	Lack of staffing to perform to perform US				
H	Lack of time to perform ultrasound				
I	Ran out of supplies of paper towel				
J	Problems with image quality				
K	The screen was not working.				
L	Lack of gel				
M	Poor quality of gel				
N	Lack of network services)				

O	Trouble with opening/using the Butterfly app				
P	Forgetting username and password				
Q	Lack of data/wifi				
R	Difficulty in the setting of scan type				
S	Difficulty with scan type Obs 1 – (Gyne & 1st trimester)				
T	Difficulty with scan type Ob2 –(2nd & 3rd trimester)				

-

Q401. If you have had any of the problems with the functioning of the POCUS equipment, was the problem solved

Yes >Q401a
No >Q403

Q401a If yes, how long did it take for the problem to be fixed

- On the same day
- Within the week
- Within the month
- It’s still broken/not working
- Others (Specify)…

Q402 If the problem was fixed, who fixed it?......................

Q403 Please rate the training along the following criteria (A Likert scale with Very good, good fair, poor)

Q403a: Pre-training preparation

Q403b. General organization

Q403c. Training venue

Q403d. Training materials content

Q403e. Duration of training

Q403f. Trainers

Q403g. Practical venues

Q403h. Practical content

Q404 Do you have recommendations for improvement of POCUS project?

Q405: If yes which area would you recommend (free text)…

a) ……………………………………………………………………………

Q406 Please feel free to include questions or comments on any questions in the space below:

