# Supplementary material for: Evaluation of large-scale implementation of obstetric point of care ultrasound in eight counties in Kenya using RE-AIM framework
Source: BMC Health Serv Res. 2025 Aug 1;25:1016. doi: 10.1186/s12913-025-13212-8 (PMC12315356; doi:10.1186/s12913-025-13212-8)
Supplement: Supplementary file 2 — Supplementary Material 2 [file 12913_2025_13212_MOESM2_ESM.pdf]

EXIT QUESTIONNAIRE FOR PRENATAL MOTHERS

Section 1: Facility Level Identification

This section is to be completed for each [Client interviewed] visited.

**Date of visit:**		**Time starts**		**Time ended:**	
**Serial #**					
Telephone number of the Participant (For follow-up purposes):					
ANC #					
Patient initials/unique no.					
gravida					
Parity					
**Q101:**	**Select the county** (*Drop down list of the 8 counties)*	Baringo--------------1<br>Kakamega-----------2<br>Kilifi----------------- 3<br>Kitui------------------4<br>Nakuru---------------5<br>Samburu-------------6<br>Taita Taveta---------7<br>Turkana------------- 8<br>Others (Specify):…9			
**Q102**	Select the Sub-County<br>*(drop down list of all)*				
**Q103:**	**Health Facility name:**	List of sampled facilities per sub-county *(in a drop list)*			
**Q104:**	**Health Facility**	Level 2			

	**Level**	Level 3<br>Level 4<br>*(List of levels in a dropdown list)*
**Facility Location**	**Urban**<br>**Rural**	

General Background information about the Participant/respondent				
First, I will ask some general questions		*CATEGORY*	*CODE*	*SKIP*
Q105	What is your age?	---------------	1<br>2<br>3<br>4	
Q106	What is the highest level of education you've completed?	None…………..<br>Primary. . . . .<br>Secondary<br>College<br>College (Middle Level.<br>University	0<br>1<br>2<br>3<br>4<br>5	

Q 107	What is your occupation	House wife<br>Professional (*for professional careers like teaching, nursing, banking, etc)*<br>Business, (specify type)<br>Small scale<br>Large scale<br>Any other, specify, ……..	1<br>2<br>3<br>4<br>9	
Q108	Marital status	Single<br>Married<br>Widow<br>Other, specify….	1<br>2<br>9	
Q109	Do you, or your partner have NHIF or any other insurance	Yes………proceed<br>No………proceed	1<br>2	
Gravida	How many times have you been pregnant including current pregnancy			
Parity1	Parity1: How many babies have you delivered after seven months onwards,			
Parity2	Parity2: How many babies are alive?			
Parity3a	Parity3: Have you lost a pregnancy?	1 Yes<br>2 No		
Parity3b	At how many weeks did you loose the pregnancy?			
Parity4	Parity4: Have you lost a baby within the 28 days after birth?	1 Yes<br>2 No		

SECTION 2: ABOUT THE CURRENT/IMMEDIATE PREGNANCY				
Q110	When was the first day of your last monthly period (LMP (*refer to ANC records or booklet) (specific date)*	____________		
Q111	Expected date of delivery (EDD) *(give specific date)*	_____________		
Q 112	Number of weeks into the pregnancy<br>(Calculate or request to refer to the antenatal) (ANC) booklet)	1-89-1617-24<br>------------------- drop down list (to 1-40 weeks)		
Q113	How many weeks into your pregnancy did you make your first antenatal visit	-------------------drop down list (1-40 weeks)<br>Drop Down		
Q114	How many antenatal visits have you had in your current pregnancy?	1<br>2<br>3<br>4<br>Other, specify ………		
Q115	Is this the first hospital you came to for your antenatal follow-up?	Yes, (*skip to Q120*)<br>No >Continue,	1<br>2	
Q116	If no, Where did you seek antenatal care services the first time?	A level …..Government facility(drop down level 1-4 hospital)<br>Mission hospital ….<br>Private facility ….<br>Any other, specify …	1<br><br>2<br>3<br>9	

Q117	Why did you move from the previous health facility to this one	Cost of the services was high ….<br>Ultra sound services were not being offered ….<br>Convenience of access …<br>Quality of services …<br>Othet Specify	 1<br><br>2<br>3<br>4	
Q120	During your current pregnancy, have you had an Ultra sound scan done?	Yes…>Q120a<br>No ….>Q120c	1<br>2	
120a	If yes, Specify the type			
120b	How may weeks was ultrasound done?	Weeks Dropdown		
Q120c	How many U/S scans have you had during the current pregnancy	use a drop down(0-10 scans)		
Q120d	At how many weeks/ into your pregnancy did you get your U/S done?	Use a drop down (1-40 weeks		
Q121a	Were you offered the US in this hospital?	1. Yes >Q121b<br>2. No >Q121c		
Q121b	In what department in the hospital did you have the ultrasound done?	At the antenatal clinic …<br>In the Maternity ward …<br>In Radiology department …<br>In a facility outside the hospital …<br>Others (Specify)…	1<br>2<br>3<br><br>4<br>9	
Q121c	Specify which hospital the US was done?			
Q123	Did the care provider performing the U/S ask for your permission before doing the U/S scan	Yes<br>No	1<br>2	

Q 124	Did the health care provider performing the ultrasound scan explain the procedure before doing it?	Yes<br>No	1<br>2	
Q125	Were you shown the U/S screen as the ultrasound scan was being done?	Yes<br>No	1<br>2	
Q126	Did the ultrasound procedure cause you any discomfort?	Yes, >Q127<br>No, > Q128	 1<br>2	
Q 127	Could you specify what the discomfort was	There was no privacy<br>Pain during the procedure<br>Other, specify…….	1<br>2<br>3	
Q128	Were you provided with a written report of the Ultrasound findings recorded?	Yes >Q129<br>No, > Q132	1<br>2	
Q129	What type of report (record) did you receive?	Report was written in the antenatal booklet …<br>It was documentation on a referral form ….<br>Any other, specify ……………….<br><br>I do not know	 1<br><br>2<br>3<br>4	
Q132	Did the clinician discuss the U/S findings with you?	Yes …. >Q133<br>No…. > Q134		

Q133	What were you told as the findings of the U/S<br>*(Refer/ confirm with the ANC booklet)*	The baby is okay<br>I have twins/ more<br>The baby heart rate had a problem<br>The placenta was not in the right position<br>The way the baby is lying would make delivery challenging.<br>The quantities of fluid in the uterus was not adequate/ was too much (*underline best descriptor*)<br>I did not understand…	1<br>2<br>3<br>4<br>5<br>6<br>7<br>8	
Q134	Have you been referred to this facility for ANC?	Yes………………<br>No……………<br>Don't know…	1<br>2<br>9	
Q135	Have you been referred to deliver in this facility?	Yes………………<br>No……………<br>Don't know…	1<br>2<br>9	
Q136	Were you referred based on the ultrasound examination?	Yes……………<br>No……………<br>Don't know……	1<br>2<br>9	
Q137	During the current pregnancy, have you experienced any complication	Yes……………<br>No……………<br>Don't know…	1<br>2<br>9	
Q138	Kindly specify the complications if known<br>(Refer to the booklet)	___________________		
9Q139	Was the complication diagnosed using POCUS during your routine ANC visits?	Yes……………<br>No……………<br>Don't know…	1<br>2<br>9	

Q140	In this pregnancy, how many babies do you carry?	One<br><br>Twins<br><br>Don't know………	1<br><br>2<br><br>9	
Q141	If twins, at what gestation was it diagnosed? (In months)<br><br>*(Refer to booklet)*	-------------		
Q142	Was it diagnosed during POCUS ultrasound examination?	Yes…………<br>No……………<br>Don't know…	1<br>2<br>9	
Q145	What did the provider tell you about the results of the ultrasound?	Everything was normal………*go to Q147*<br><br>Something was not as it should be……<br><br>Cant Remember………	1<br><br>2<br><br>3	If Q145=2, should see Q146, Q148, Q149<br>If Q145=1, should see Q149<br>If Q145=2, should see Q149
Q146	Did the health care provider explain what wrong?<br><br>(Refer to booklet)	Yes, …………………………………….<br>No		

Q148	If yes, what were you told to be the problem?<br><br>(Check the booklet for records)	The placenta positions<br><br>That I am carrying more than one baby<br><br>The baby is sitting with it buttocks<br><br>The baby's heart rate was too slow/fast (*underline)*<br><br>The fluid in the uterus was too much/too little (*underline*)<br><br>Others (Specify)…………		
Q149	Did you trust the results of the US?	Fully<br>To Some extent<br>Not at all		
Q151	Did you fear there could be any risk associated with undergoing an ultrasound exam?	Yes…>Q152<br>No…..>Q153	1<br>2	
Q152	If yes, what did you fear.	Risk to unborn baby<br>Risk to my health<br>Risk to both mother and unborn baby<br>Risk of a miscarriage<br>Any others, specify ………………		

Q153	Did your feelings towards your baby change after doing the ultrasound?	Yes……>Q154<br>No…….>Q155	1<br>2	
Q154	Please explain how doing the ultrasound affected your feelings towards your baby	Positive feeling<br>Negative feeling<br>Neutral *(it had no effect)*<br>*Any other, specify, ……*		1<br>2<br>3
Q155	How did you feel after seeing the scan on the screen during the ultrasound exam? - Provide the scale	Happy ……<br>Unhappy ……<br>Anxious …….<br>Not sure …..<br>Any, other, specify …..		1<br>2<br>3<br>4<br>5
Q 156	How long did you have to wait during your ANC visit today?	Less than 30 minutes<br>31-60 minutes<br>Over 60 minutes		1<br>2<br>3
Q157	Was Ultrasound part of the antenatal review or you needed to wait for it separately	It was included in the routine reviews …<br>I needed to wait separately for the ultrasound …		1<br>2
Q158	If the ultrasound scan was done separately, how long did you have to wait for the US component?	0-15 minutes<br>16-30 minutes<br>30-45 minutes<br>More than 45 minutes, specify, ….		1<br>2<br>3<br>4
Q159	Did you pay for the ultrasound exam done using the POCUS machine? *(show the picture of Probe and Ipad)*	Yes >Q160<br>No, >Q161		1<br>2

Q160	If yes how much did you paid (Ksh.)?	1 - 1000 Ksh.<br>1001-3000kshs<br>More than 3000Kshs<br>Don't k now ….	
Q161	How would you rate the quality of care you received today?	Very poor<br>Poor<br>Good<br>Very good<br>Excellent…	1<br>2<br>3<br>4<br>5
Q162	How satisfied are you with the care you received today?	Not satisfied at all<br>Not satisfied…<br>Neutral……<br>Satisfied……<br>Very satisfied…	1<br>2<br>3<br>4<br>5
Q163	Please explain why you rate the services offered this way: (Free text)		
Q164	Would you come back for a similar ultrasound?	Yes… > Q 166<br>No……>Q165	1<br>2
Q165	If no, please explain why not	________________________________	
Q166	How likely would recommend this facility to a family member or friend, if they were pregnant?	Extremely unlikely<br>Somewhat unlikely<br>Neutral<br>Somewhat Likely<br>Extremely likely	1<br>2<br>3<br>4<br>5

Q168	How likely would recommend the POCUS scan to other pregnant women?	Extremely unlikely<br>Somewhat unlikely<br>Neutral<br>Somewhat Likely<br>Extremely likely	1<br>2<br>3<br>4<br>5
168	How likely are you to recommend the POCUS scan to other pregnant women?	Extremely unlikely<br>Somewhat unlikely<br>Neutral<br>Somewhat Likely<br>Extremely likely	1<br>2<br>3<br>4<br>5

SECTION 3: OBSTETRICS HISTORY FOR PRIOR PREGNANCIES

I will ask some questions about previous pregnancy/ies		***CATEGORY***	***CODE***	***SKIP***
Q170	For your previous (last) pregnancy *(if applicable),* was an ultrasound done?	Yes…>Q170a<br>No…>Q1703<br>Don't know >Q173		
Q170	At how many months of that pregnancy was the ultrasound done in your last pregnancy?	Within the first 3 months …..<br>Between the 3rd and 6th month…<br>Close to date of delivery 1…..		1<br>2<br>3
Q171	For ultrasound done in your last pregnancy, what was the reason?	I requested for it …….<br>A clinician recommended that I needed one………<br>I don't know ………<br>Others (Specify) …………….		1 *Go to Q173*<br>2<br>3<br>4

Q173	Have you had any of the following conditions with previous pregnancies? (Select all that apply)	Twins/multiple gestation (more than one baby).<br>Breech delivery (baby in sitting position)<br>Preterm birth<br>Placenta problem<br>Caeserian section (Operation for birth)<br>Other (Specify).....<br>None of the above	1<br>2<br>3<br>4<br>5
