# Supplementary material for: Evaluation of large-scale implementation of obstetric point of care ultrasound in eight counties in Kenya using RE-AIM framework
Source: BMC Health Serv Res. 2025 Aug 1;25:1016. doi: 10.1186/s12913-025-13212-8 (PMC12315356; doi:10.1186/s12913-025-13212-8)
Supplement: Supplementary file 3 — Supplementary Material 3 [file 12913_2025_13212_MOESM3_ESM.pdf]

APPENDIX 3: QUESTIONNAIRE FOR POSTNATAL MOTHERS

Section 1: Facility Level Identification

This section is to be completed for each [Client interviewed] visited.

**Date of visit:**		**Time start**		**Time ended:**	
**RA Unique ID**					
Telephone number of the Participant (For follow-up purposes):					
**Q1: County** *(Circle the appropriate response)*	Baringo--------------1<br>Kakamega-----------2<br>Kilifi-----------------3<br>Kitui------------------4<br>Nakuru-------------5<br>Samburu------------6<br>TaitaTaveta--------7<br>Turkana-------------8	**Q2: Sub-County:**<br>______________________________			
**Q3: Health Facility name:**		**Q4: Health Facility Level**			

General Background information about the Participant				
First, I will ask some general questions		*CATEGORY*	*CODE*	*SKIP*
Q5	What is your age in years?	___________________		
Q6	What is the highest level of education you have completed?	None..............<br>Primary. . . . .<br>Secondary<br>College<br>University	0<br>1<br>2<br>3<br>4	
Q7	What is your employment status?	Employed<br>Self employed<br>Unemployed		

Q8	What is your marital status	Single<br>Married<br>Widow<br>Separated<br>Divorced	1<br>2<br>3<br>4<br>5	
Q9	Do you have, or your partner has NHIF/ Linda mama or any other medical insurance?	Yes……………………<br>No……………………	1<br>2	
**ULTRASOUND RELATED INFORMATION**				
Q 10	Have you had an ultrasound during this pregnancy? If yes go to Q10a	Yes >Q10a<br>No>Q13		
Q10a	What type of ultrasound was performed. if POCUS (provide POCUS picture)<br><br>Q10b. Do you have a printout or not? Or receipt)<br>1 Yes<br>2 No<br>POCUS should be free with no printout.	• POCUS<br>• OTHER<br>• Don't Know		
Q11	When was the first ultrasound done? (Gestational weeks)<br>[*confirm with ultrasound if available*] refer to booklet and compare with last menstrual period during the *ultrasound*)	0-12 weeks<br>13 to 24 Weeks<br>25-37 Weeks<br>38-41 Weeks<br>Don't know		

Q12	Where was the ultrasound done?<br>[*Name place where the ultrasound was done*]	Antenatal Clinic,<br>Maternity,<br>Radiology department<br>Outside the facility		
Q13	Were you referred to deliver in this facility?	Yes………..>Q13a<br>No…………..Q13c	1<br>2	
Q13a	If yes, where were you referred from?			
Q13b	The level of facility referred from			
Q13c	Were you referred based on the ultrasound examination? If yes go to Q13d	Yes >Q13d<br>No >Q13f<br>Don't know…… Q13f	1<br>2<br>3	
Q13d	If yes, was it a POCUS Ultra sound?	1. Yes<br>2. No		
Q13e	What was the reason for referral?	I don't know why I was referred<br>I wanted to deliver at this facility<br>I was told I needed a caesarian section<br>I was told I had a complication and could not be delivered as originally planned<br>Other, specify……		
Q13f	When did you deliver this baby/s?	Indicate date of birth		
Q14	How did you deliver<br>(Confirm from history in the booklet) If caesarian section go to Q14a	Vaginal delivery (SVD)<br>Cesarian Section (Operation)……<br>Vaginal breach....<br>Don't know……….	1<br>2<br>3	

Q14a	What was the reason for Cesarian Section (check the notes/book)	………………………		
Q14b	What is the outcome of birth (Newborn status)	Live newborn<br>Stillbirth/s		
Q14c	How many baby(s) did you give birth to	One baby<br>More than one…. Go to Q14d		
Q14d	What was the birth weight for the baby(s)	First newborn<br>Above 2500g<br>1501g<2500g<br>1000-1500g<br>Below 1000<br><br>Second newborn<br>Above 2500g<br>1501g<2500g<br>1000-1500g<br>Below 1000<br><br>Third newborn (optional)<br>Above 2500g<br>1501g<2500g<br>1000-1500g<br>Below 1000		

Q14e	What was the APGAR score for the baby(s) (refer to the booklet)	First Newborn<br>○ 1 Minutes:<br>○ 5 minutes<br>○ 10 minutes<br>Second Newborn :<br>○ 1 Minutes:<br>○ 5 minutes<br>○ 10 minutes<br>Third Newborn :<br>○ 1 Minutes:<br>○ 5 minutes<br>○ 10 minutes<br>○ Didn’t give birth to third child		
Q14f	What was the gestational age(weeks) for the just completed birth			
Q19	When the latest POCUS was done for the current pregnancy, what did the provider tell you about the results of the ultrasound?	Everything was normal………<br>Something was abnormal……<br>Other, specify…..<br>Don’t know………		
Q20	Did you trust the results of the ultrasound?	Yes<br>No		
Q20a	How did you feel about the POCUS ultrasound exam?	I did not feel anything<br>A little excited<br>Moderately excited<br>Very much excited<br>Extremely excited		

Q21	Were you shown the images on the ultrasound screen?	Yes……………<br>No………	1<br>2	
Q22	Did you receive an explanation of the ultrasound screen images?	Yes………<br>No………………	1<br>2	
Q23	Did you have any fear during the ultrasound exam?	Yes…………………………<br>No…………………………	1<br>2	
Q24	Did you fear any risk associated with ultrasound exam?	Yes……………>Q24a<br>No………..>Q25	1<br>2	
Q24a	If yes, what did you fear?			
Q25	Did your feelings towards your baby change after seeing the ultrasound images? If yes go to Q26	Yes……………>Q26<br>No…………….>Q29	1<br>2	
Q26	Please explain how seeing the ultrasound affected your feelings towards your baby	______________________________<br>______________________________<br>______________________________<br>_____________________		
Q28	How much did you pay for the ultrasound scan for this pregnancy?	0 kshs<br><br>1000-3000kshs<br><br>More than 3000Kshs<br><br>Don't k now….		
Q29	Would you come back for another POCUS ultrasound?	Yes…… >Q30<br>No………>Q29a	1<br>2	
Q29a	If no why			

Q30	How likely or unlikely is it that you would recommend this facility to a family member or friend, if they were pregnant?	Extremely unlikely<br>Somewhat unlikely<br>Neutral<br>Somewhat Likely<br>Extremely likely	1<br>2<br>3<br>4<br>5	
Q35	During the previous pregnancy was ultrasound ever done?	Yes<br><br>No		

SECTION 2: OBSTETRICS HISTORY				
First I will ask some questions around pregnancy history		*CATEGORY*	*CODE*	*SKIP*
Q45	What is the number of total children you have including the current delivery? [write in numbers]	______________		
Q46	What was the outcome of the previous pregnancy before this just ended birth?	Miscarriage……….<br>Preterm……………….<br>Full-term………<br>Don't know………	1<br>2<br>3<br>9	
Q47	Have you had any of the following conditions with other pregnancies? (Select all that apply)	Twins/multiple gestation<br>Breech delivery (sitting position)<br>Preterm birth<br>Placenta problem<br>Caesarian section (operation for birth)<br>Other (Specify).....<br>None of the above		1<br><br>2<br>3<br>4<br>5<br>6<br>7

SECTION 3: ABOUT THE CURRENT/IMMEDIATE PREGNANCY

		CATEGORY	CODE	SKIP
Q48	How many times did you attend antenatal clinic with this pregnancy?	0<br>1<br>2<br>3<br>4<br>4+		
**SECTION 5: MATERNAL OUTCOME**				
Q49	After delivery, did you develop any complications personally? If yes Q50	YES . . . .<br>NO . . . .<br>Don't know ……		
Q50	Please specify the type of complications that you experienced (Double check the register /file/booklet to confirm any complications/outcomes)			

Please feel free to include questions or comments on any questions in the space below:

Thank the mother and end the interview.
