# Supplementary material for: Evaluation of large-scale implementation of obstetric point of care ultrasound in eight counties in Kenya using RE-AIM framework
Source: BMC Health Serv Res. 2025 Aug 1;25:1016. doi: 10.1186/s12913-025-13212-8 (PMC12315356; doi:10.1186/s12913-025-13212-8)
Supplement: Supplementary file 4 — Supplementary Material 4 [file 12913_2025_13212_MOESM4_ESM.pdf]

HEALTH FACILITY CHECK LIST

**Date of visit:**		**Time**	
**County:**		**Sub-County:**	
**Health Facility name:**		**Health Facility Level**	
**Facility Type** *(circle the type of facility)*	1. Government	**Site of Facility** *(Circle the site of facility)*	**1. Urban**<br>**2. Rural**

**RE-AIM COMPONENT**	**DATA**	**Response**	**Comment**
**REACH**			
	Staffing: (in general?): Total number of:<br>a) Nurses:-----------------------------------<br>b) Doctors: --------------------------------<br>c) Clinical Officers:--------------------------<br>d) Radiographers:--------------------------		
	Staffing: (ANC, labour ward): Total number of:<br>a) Nurses:-----------------------------------<br>b) Doctors: -----------------------------------<br>c) Clinical Officers:-------------------------<br>d) Radiographers:--------------------------		
	Staff trained on POCUS<br>a) Nurses:-----------------------------------<br>b) Doctors: -----------------------------------<br>c) Clinical Officers:-------------------------<br>d) Radiographers/sonographers:--------------------------		
	Current deployment of Staff trained on POCUS<br>a) MNCH:-----------------------------------<br>b) Labour ward: ------------------------------------<br>c) General OPD:-------------------------<br>d) Other wards		

**EFFECTIVENESS &ADOPTION**	Other staff trained on POCUS through on Job-training<br>a) Nurses:-----------------------------------<br>b) Doctors: -----------------------------------<br>c) Clinical Officers:-------------------------<br>d) Radiographers		
	Number of Butterfly probes in the facility (please confirm)		
	Number of Butterfly probes in use the facility (please confirm)		
	Where is/are the Butterfly probe and Ipads stored? (please confirm)		
	Number of antenatal mothers seen per Month		
	Number of deliveries per month		
	Number of referrals to other facility(ies) per month--------<br>(*Indicate reasons for referral)*		
	Number of referrals from other facility(ies) per month		
	Number of live births per month--------------		
	Number of still births per month (Indicate whether fresh or macerated		
**Implementation**	Is there availability of electricity or solar to charge the probe and the Ipad		
	Is gel available or sourced by the facility		
	Where is the gel stored		
	Are paper towels for wiping the gel off availability where the POCUS is done		
	Where was the probe located when you arrived		
	Was this the usual location		

	Are there lockable cabinet for storage of the POCUS equipment & supplies		
	Where are the POCUS examinations done?		
**Maintenance**	In what condition did you find the US on arrival<br>a. Good working condition and clean<br>b. Not charged<br>c. Not clean		
	Is there Dedicated room for POCUS e.g. room, couch, linen towels, gel to maintain POCUS		
	Is there an allocation of funds to buy linen towels for POCUS		
	Is there an allocation of funds to buy gel for POCUS		
	Are there other staff willing to be trained on POCUS in the facility		
	Are there staff trained on POCUS who have been transferred?		
	If yes, did they go with the probe to where they were transfered		
	Are they tracking data on POCUS right now and reporting to in-charge of facility or County health department		
