# Supplementary material for: Evaluation of large-scale implementation of obstetric point of care ultrasound in eight counties in Kenya using RE-AIM framework
Source: BMC Health Serv Res. 2025 Aug 1;25:1016. doi: 10.1186/s12913-025-13212-8 (PMC12315356; doi:10.1186/s12913-025-13212-8)
Supplement: Supplementary file 6 — Supplementary Material 6 [file 12913_2025_13212_MOESM6_ESM.pdf]

KEY INFORMANTS IN-DEPTH INTERVIEW GUIDE

Introduction:

Thank you for agreeing to take part in this interview. Before we begin, I would like to clarify some information with you. With your permission, the interview will be recorded. There are no ‘right’ or ‘wrong’ answers to the questions I will ask you. I am interested in your opinions and experiences of the Point of Care Ultrasound (POCUS) training and implementation you were involved in. I will take out the names of anyone you mention during the interview to protect their identity. If there are any questions that you do not wish to answer, please tell me and we will move to the next question. If you wish to pause or stop the interview at any time, again, please let me know. Are there any questions that you would like to ask me about the interview before we begin?

Are you happy to proceed? And for the interview to be recorded?

Biographical data:

1. Institution______________
2. Department_________
3. Gender-----------------
4. Age-------------------
5. Designation______________
6. Position…………………..
7. Duration at current position____________
8. Total years of professional experience_______________

Introductory questions

Tell us about how you were specifically involved in the POCUS project (e.g., training, hospital leadership, etc).

REACH

1. How many of your staff were trained in POCUS?
2. How have you deployed them after training? (e.g. to what units)
3. From what you have observed, do trained providers conduct scans regularly?
    a. What is the average number of POCUS examinations being done per day/week/month/quatre?
    b. Do you have any records showing the number of women who have received POCUS (for example - daily/weekly/monthly reports)

EFFECTIVENESS

1. In your view, how has POCUS impacted the quality of care antenatally and during labour?
2. Are you aware of any documentation being done by the HCW of their findings of POCUS?
    1. Do you have records of women that have been referred to other facilities based on the findings of POCUS?

ADOPTION (COUNTY/FACILITY)

1. How in your opinion does POCUS impact the experience of the health care providers while managing expectant woman during the antenatal period and during labor?
2. In your opinion, how does offering POCUS impact the demand from women (e.g. ANC attendance)?
    a. Influence of the POCUS program so far within the county/facility
    b. Behaviour of recipients and providers towards the program

IMPLEMENTATION

1. What in your opinion has supported the implementation of POCUS?
    a. Are there any available SOP/ guidelines related to POCUS?
2. .What challenges if any could be affecting the smooth implementation of POCUS?
    a. Supplies, staffing issues/turnover
3. How is your office involved in managing security and access to the equipment?
    a. How is safety of probe ensured (entered into the hospital inventory, are they all in use or as some locked up)?

MAINTENANCE

1. Since POCUS has been provided free, tell me your opinion in sustaining the services?
    a. Probes: have they been charging? any intention to charge?
2. What strategies have you put in place in your facility (county/sub-county) since the introduction of POCUS to support , maintain the momentum of the program, or to ensure its sustainability?
    a. *Committed to allocating dedicated funds for supplies, supportive supervision and further training , trained staff retention, willingness to facilitate more staff for training, policies/guidelines/SOPs, equipment maintenance (Butterfly).*
    b. Resources from o*ther implementing partners, such as* supportive supervision/mentorship, continuous professional development (CPD) (GUSI, Butterfly/facility)
3. What recommendations do you propose for continued implementation?
    a. *Committed to allocating dedicated funds, trained staff retention, willingness to facilitate more staff for training, policies/guidelines/SOPs, equipment maintenance (Butterfly), collaborative services with radiology, other partners, and private sector.*

Closing Comments

Thank you so much for taking your time to participate in this interview today.
