# Supplementary material for: Evaluation of large-scale implementation of obstetric point of care ultrasound in eight counties in Kenya using RE-AIM framework
Source: BMC Health Serv Res. 2025 Aug 1;25:1016. doi: 10.1186/s12913-025-13212-8 (PMC12315356; doi:10.1186/s12913-025-13212-8)
Supplement: Supplementary file 7 — Supplementary Material 7 [file 12913_2025_13212_MOESM7_ESM.pdf]

APPENDIX 7: MOTHERS' QUALITATIVE DATA COLLECTION INTERVIEW GUIDE

Thank you for agreeing to take part in this interview. Before we begin, I would like to clarify some information with you. With your permission, the interview will be recorded. There are no 'right' or 'wrong' answers to the questions I will ask you. I am interested in your opinions and experiences of the ultrasound examination done (where the nurse or doctor or radiographer used the machine to see your baby inside your body). I will take out the names of anyone you mention during the interview to protect their identity. Your participation will not affect the care you receive at this facility. If there are any questions that you do not wish to answer, please say and we will move to the next question. If you wish to pause or stop the interview at any time, again, please let me know. Are there any questions that you would like to ask me about the interview before we begin?
Just to remind ourselves, sometimes I will refer to ultrasound as "POCUS" or point of care Ultrasound.
Are you happy to proceed? And for the interview to be recorded?

a) Maternal Characteristics Check the booklet for ANC to extract maternal characteristics

How old are you (years)?

ANC woman	Postnatal women
ANC no.<br>Gravidity<br>Parity<br>LMP<br>EDD<br># weeks gestation	PN no<br>Parity<br>Gestational age at birth<br>Birth weight<br>Neonatal outcome/complications (e.g., multiples, CS, stillbirth, etc).

What is your occupation?

What is the highest level of school you completed?

1) No formal education
2) Primary
3) Secondary
4) College and above

What is your marital status now?..................................

1) Single
2) Married
3) Widowed

How many children do you have?

EXPERIENCES DURING PREGNANCY

Tell me about your pregnancy and/birth journey

Probe: What is a smooth pregnancy? Have you experienced any problems with your pregnancy?

REACH

When did you first receive ultrasound and what was the reason? (Check the booklet)

Probes: scan during ANC or maternity unit; recommended by provider; personal desire

<u>EFFECTIVENESS</u>

What did they find after the US? (Check the booklet and/or medical record

Probes: normal, any complications; referral

<u>ADOPTION</u>

<u>What were the reasons you decided to get scanned?</u>

Probes: recommended by provider; personal desire; free of cost, not given a choice

<u>Did you have any feelings that made you not want to get scanned?</u>

Probes: fear, cost, husband did not want; what did provider do to make you feel more comfortable getting scanned

Have you had an ultrasound scan before this?
Probes: How would you compare the two scans? *Probe on Comfort, cost, reporting?*

IMPLEMENTATION

What was your experience during the ultrasound and the service provided to you?

Probes: What did the clinician tell you before and during and after the scan? Did they clinician doing the POCUS ask for permission before performing the procedure? Did they explain to you how it works, and what you might feel during the US? Were you in a private setting where you felt comfortable? Where you asked to pay?

How did receiving POCUS make you feel about you, your pregnancy and your baby? Was anybody with you when the scan was performed, Who? How was that helpful or not?

Probes: Comfort in knowing your baby was healthy; scared, unsure what to do?

MAINTENANCE

How important do you consider a scan to be during pregnancy, and labor?

Would you recommend POCUS to a friend or family member?

How much would you be willing to pay for the type of scan you did with a POCUS machine?

Closing question
Is there anything else about the portable ultrasound services that we haven't discussed and would like to tell me about?

Closing Comments

Thank you so much for taking your time to participate in this interview today.
